# Antimicrobial Efficacy of Probiotic and Herbal Oral Rinses against *Candida albicans* in Children: A Randomized Clinical Trial

**DOI:** 10.5005/jp-journals-10005-1328

**Published:** 2016-04-22

**Authors:** Rahul Mishra, Shobha Tandon, Monika Rathore, Molay Banerjee

**Affiliations:** 1 Assistant Professor, Department of Dentistry, UP Rural Institute of Medical Sciences and Research, Saifai, Uttar Pradesh, India; 2 Ex-Dean, Department of Pedodontics and Preventive Dentistry, Babu Banarasi Das College of Dental Sciences, Lucknow, Uttar Pradesh, India; 3 Professor, Department of Pedodontics and Preventive Dentistry, Babu Banarasi Das College of Dental Sciences, Lucknow, Uttar Pradesh, India; 4 Professor and Head, Department of Microbiology, Babu Banarasi Das College of Dental Sciences, Lucknow, Uttar Pradesh, India

**Keywords:** Candida albicans, Chlorhexidine digluconate, Dental caries, Herbal oral agent, Probiotics.

## Abstract

**Background:** A growing number of dentists are embracing the philosophy that natural agents are better for children’s oral health. Knowledge of probiotics on host immune system has entered a new phase of research, and progression in this field is likely to offer novel means by modulating host immunity for prevention and treatment of a wide variety of oral diseases.

**Aim:** To compare the antimicrobial efficacy of probiotics in reducing salivary *Candida albicans* counts with commonly used antimicrobial agents like 0.2% chlorhexidine and herbal rinse.

**Materials and methods:** A randomized clinical trial was conducted on 60 subjects aged between 6 and 14 years, for a period of 9 months. The subjects were randomly divided into three groups comprising 20 subjects in each group. Three oral agents were administered twice daily for a maximum period of 1 week. *Candida albicans* counts were recorded before and after intervention and the results were submitted for statistical analysis using Statistical Package for the Social Sciences (SPSS) version 15.0 software.

**Results:** The change in mean log_10_ colony-forming unit (CFU)/ ml of *C. albicans* in groups A to C was 0.43 ± 0.72, 0.68 ± 1.05 and 0.22 ± 0.66 CFU/ml respectively.

**Conclusion:** Data obtained from the study demonstrated that probiotic rinse was equally effective as 0.2% chlorhexidine digluconate rinse in reducing *C. albicans* counts after 1 week of intervention. Herbal oral rinse was least effective. Probiotic oral rinses have opened new horizons in improvement of oral health by maintaining healthy ecosystem. However, a longitudinal study with larger sample size needs to be undertaken to evaluate the therapeutic effects of probiotics and herbal agents.

**How to cite this article:** Mishra R, Tandon S, Rathore M, Banerjee M. Antimicrobial Efficacy of Probiotic and Herbal Oral Rinses against *Candida albicans* in Children: A Randomized Clinical Trial. Int J Clin Pediatr Dent 2016;9(1):25-30.

## INTRODUCTION

*Candida albicans* is the most frequently isolated fungal species in children. It has the ability to colonize the tooth surface and invade dentinal tubules. Growth and colonization of *C. albicans* is favored by change in homeostasis of oral cavity, prolonged intake of antibiotics or alteration in host’s defense mechanism, which results in transformation of saprophytic form of *C. albicans* to parasitic form.^1^

Akdeniz et al^2^ found that prevalence of *Candida* species was 69.2% in children with decayed teeth and 5% in caries-free subjects. Timely intervention is needed to prevent exacerbation of carious lesions and colonization to other sites.

Various studies utilizing chemical products for the treatment of oral diseases have reported significant reduction in pathogenic microbiota without encouraging growth of opportunist pathogens in the oral cavity.^3^ Chlorhexidine digluconate, a gold standard in the field of dentistry, is a potent broad-spectrum antimicrobial agent with advantage of substantivity.^3^ However, few individuals find its taste bitter; it causes light-brown stain on tooth and so affects the esthetics and there is loss of sensitivity of taste for hours after rinsing.^4^ These side-effects limit long-term use of chlorhexidine as rinse and its acceptability by patients. Therefore, the search for a new rinse continued and the scientific focus shifted toward biogenic agents for the prevention of oral diseases.^3^

Since times immemorial, herbal hygiene measures have been practiced by different populations around the world. Evolution of Ayurveda and plant-based remedies for treatment of various disorders and to maintain good health through day-to-day life experiences is a part of the cultural heritage of India and is now practiced in the rest of world as alternative or complementary medicine. No side effects of any herbal agents have been reported till date. Apart from this, due to the absence of alcohol and/or sugar, herbal oral rinse can be preferred in high caries risk group children.

Chewing of neem sticks *(Azadirachta indica)* as a substitute for toothbrush is still practiced in rural India.^5^ Triple myrobalans ‘Triphala’ has been extensively used in Ayurveda due to its anticariogenic and antimicrobial properties.^6^ Tea tree oil used in mouthwashes and toothpastes have antiseptic and antifungal property. *Aloe barbadensis* Miller or *aloe vera,* a widely used herbal product, has antibacterial, antifungal, analgesic properties and reduces mucosal inflammation.

Besides herbal agents, several investigators have suggested the role of probiotics or friendly bacteria in oral health, by improving indigenous flora. According to the World Health Organization (WHO) (2001), ‘Probiotic refers to live microorganisms, which when administered in adequate amounts confer health benefit on the host.’^7^ For over a century, holistic approaches have been practiced to treat dysbiosis of gut. It causes modulation of bacterial population in order to maintain healthy ecosystem and also possesses an immunomodulatory activity by stimulating nonspecific immunity and modulating humoral and cellular immune response.^8,9^

Conventional approaches alone are not sufficient in those with extensive tooth decay, an additional therapy with antifungal property is needed for elimination of candidal reservoir. With increasing antibiotic resistance, it is important that we turn our attention to holistic approaches to combat infections.^10^

The role of herbal and probiotic rinses against fungal infections is poorly understood, which is quiet prevalent in young children. Hence, our present study was conducted with the aim to compare the efficacy of probiotics in children with 0.2% chlorhexidine digluconate and herbal oral rinses in reducing *C. albicans* counts.

## MATERIALS AND METHODS

The present study was conducted at the Department of Pedodontics and Preventive Dentistry in collaboration with the Department of Microbiology, Babu Banarasi Das College of Dental Sciences (BBDCODS), Lucknow, Uttar Pradesh, India. Protocol approval was obtained from the Institutional Ethics Committee, BBDCODS, Lucknow. Before commencement of the study, the purpose of the study was explained to the parents and written informed consent was obtained.

### Study Design

A randomized clinical trial was conducted on 60 subjects aged between 6 and 14 years attending the Pedodontics Outpatient Unit. Trial subjects were randomly divided into three groups comprising 20 subjects in each group. Three oral rinses were administered twice daily for 1 week with the aim to compare the efficacy of probiotics and herbal rinses in children with 0.2% chlorhexidine digluconate in reducing *C. albicans* counts.

### Inclusion Criteria

- Children with carious teeth.
- No history of antimicrobial or any drug used in the past 3 months.

### Exclusion Criteria

- Patient on any other oral hygiene regimen other than routine toothbrushing.
- Patients undergoing specialized treatment, e.g., orthodontic treatment, wearing space maintainer or any other appliance.

**Figs 1A to C:**
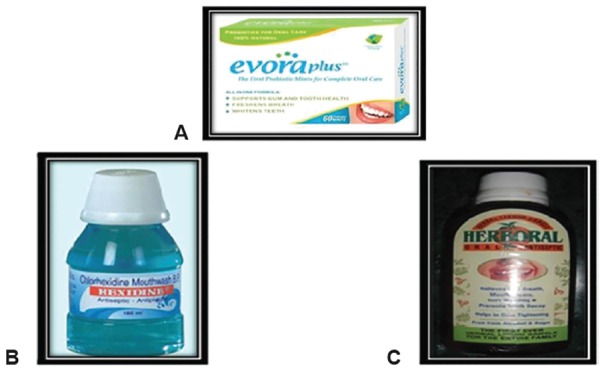
Oral agents: (A) Probiotic oral mint tablet, (B) Chlorhexidine mouthrinse, and (C) Herbal mouthrinse

The oral agents used for the present study were (Fig. 1) probiotic mint tablets (Evora Plus™, Florida, USA, composed of Probiora3), 0.2% chlorhexidine digluconate oral rinse (Hexidine®, ICPA Health Products Ltd., Andhra Pradesh, India) and herbal oral rinse (Herboral, M-Tech Innovations Ltd., Pune, India).

The study was a double blind study and the designated rinse was dispensed by the investigator to each subject in a sealed bottle without knowing the identity of the rinse.

*Group A:* Subjects were administered probiotic mint tablet by mixing the tablet with 5 ml of bottled or filtered water in a supplied measuring cup.

*Group B:* Subjects were administered 0.2% chlorhexidine digluconate oral rinse.

*Group C:* Subjects were administered herbal oral rinse.

**Fig. 2:**
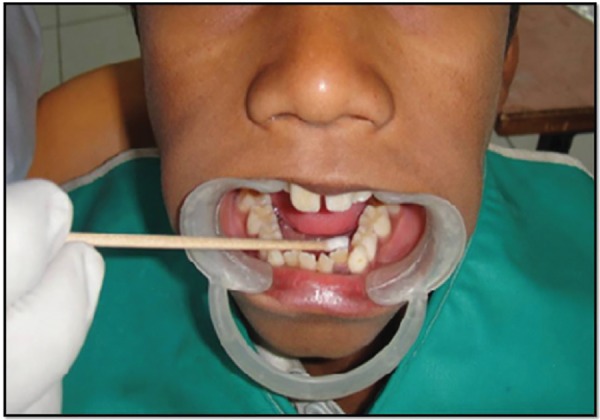
Swab technique for saliva sample

Parents were advised to administer 5 ml oral rinse twice daily half an hour after brushing in the morning and at night for a maximum period of 1 week. Furthermore, the subjects were advised to retain the rinse in oral cavity for a period of 1 minute followed by expectoration of rinse under parent’s supervision and were instructed not to consume any form of solid or liquid foodstuff for another half an hour after the rinse. The patients were constantly monitored to check whether they were following the aforementioned regimen regularly. During the intervention period, the subjects were encouraged to maintain normal oral hygiene protocol. Saliva sample was collected at baseline and after 7 days of intervention and the number of *C. albicans* colony-forming unit (CFU) per milliliter was estimated. For microbiological estimation, 2 ml of stimulated saliva was collected from each subject in a sterile vial. The vials were immediately transferred to the Department of Microbiology, BBDCODS, Lucknow, Uttar Pradesh, India, where further evaluation was carried out (Fig. 2).

**Fig. 3:**
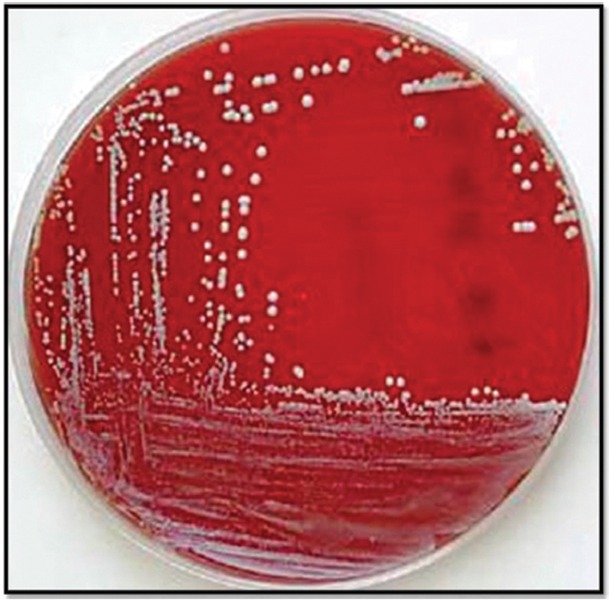
*Candida albicans* culture plate

Salivary samples were heated at 55°C for 2 minutes to disaggregate whole salivary components, and to facilitate microbial recovery it was homogenized in a vortex. A 0.1 ml sample of saliva was plated onto Sabouraud’s agar and incubated at 37°C for 72 hours. Characteristic creamy white colonies were identified and subjected to CFU/ ml count (Fig. 3). The CFU/ml count of *C. albicans* was transformed to logarithmic (Log) scale. The values in the groups were subjected to statistical analysis. Yeasts were identified on the basis of chlamydospore formation in cornmeal agar cultures at 20°C and Germ Tube formation (Reynolds Braude Phenomenon) in human serum within 2 hours of incubation period at 37°C.

### Statistical Analysis

Statistical analysis was carried out using Statistical Package for the Social Sciences (SPSS) version 15.0 software (SPSS Inc., Chicago, IL, USA). The values are represented in number (%) and mean ± SD (standard deviation). Student’s t-test (unpaired t-test) was used to assess the significance of two means of each oral rinse and paired t-test to compare the change in a parameter at two different time intervals, i.e., at baseline and after 1 week. Finally, analysis of variance (ANOVA) test was used to find the intragroup and intergroup variances among the study groups.

## RESULTS

The mean age of the subjects in group A was minimum (8.65 ± 2.01 years) followed by group B (8.90 ± 2.99 years), while in group C it was maximum (9.70 ± 3.01 years). However, on statistical evaluation, no significant difference was seen among the three groups (p = 0.410) (Table 1).

In group A, the majority of subjects were females (55%) while in group B and group C, 45 and 40% subjects were females. Despite proportional differences in gender, statistically no significant difference among groups could be seen (p = 0.626) (Table 2).

**Table 1:** **Table 1:** Agewise comparison of the three groups

*Sl. no.*		*Group*		*No. of cases*		*Mean age*		*SD*	
1		A		20		8.65		2.01	
2		B		20		8.90		2.99	
3		C		20		9.70		3.01	

F = 0.907; p = 0.410; SD: Standard deviation

**Table 2:** **Table 2:** Genderwise comparison

				*No. of*		*Females (n = 28)*				*Males (n = 32)*			
*Sl. no.*		*Group*		*cases*		*No.*		*%*		*No.*		*%*	
1		A		20		11		55		9		45	
2		B		20		9		45		11		55	
3		C		20		8		40		12		60	

χ^2^ = 0.938 (df = 2); p = 0.626

Preintervention, *C. albicans* count ranged between 0 and 1000 in the study subjects. As there was wide exponential within group variability, instead of comparing absolute values, the log_10_ values were taken for comparison. The mean log_10_ CFU/ml of *C. albicans* in group A was 1.86 ± 1.26 which was maximum followed by group B with 1.79 ± 1.35 and group C with 1.46 ± 1.39 (Table 3).

**Graph 1:**
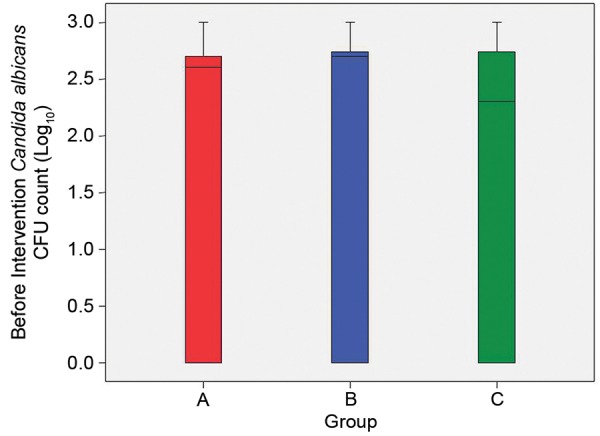
The interquartile ranges in box plot of preintervention mean colony-forming unit/ml counts of *Candida albicans* in three groups

The interquartile range as depicted in the box plot showed overlapping in a major part (Graph 1).

On comparing preintervention data by performing ANOVA, statistically no significant difference in mean log_10_ CFU/ml of *C. albicans* was seen in the three groups (f = 0.453; p = 0.638).

Preintervention pairwise multiple comparisons did not reveal statistically significant intergroup difference (p > 0.05) (Table 4).

Postintervention *C. albicans* CFU/ml count ranged between 0 and 500 in the study subjects. In all the groups the range was the same. The mean log_10_ CFU/ml of *C. albicans* in group A was 1.436 ± 1.215 which was maximum followed by group C with 1.268 ± 1.310 and then group B with 1.109 ± 1.268 (Table 5).

**Graph 2:**
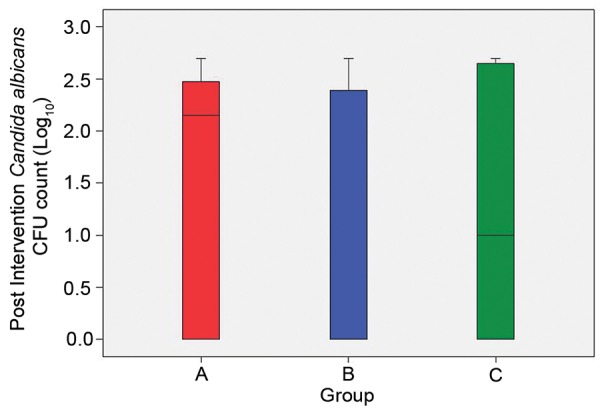
The interquartile ranges in box plot of postintervention of mean colony-forming unit/ml count of *Candida albicans* in three groups

The interquartile ranges as depicted in the box plot show major overlapping, thereby implying that the groups behaved on a similar pattern (Graph 2).

Though differences existed in groups as far as mean value was concerned, the high variability and range of results did not show consistent behavior of any of the three group. Analysis of variance revealed that despite differences in mean values, statistically the groups were not significantly different from each other (p = 0.717) (Table 6).

Postintervention pairwise multiple comparisons revealed that none of the comparisons show a significant difference (Table 7).

**Table 3:** **Table 3:** Preintervention colony-forming unit/ml count of *Candida albicans* in three groups - absolute and log_10_ values

		*Absolute count*						*Log_10_ values*					
		*Group A*		*Group B*		*Group C*		*Group A*		*Group B*		*Group C*	
Min		0		0		0		0.000		0.000		0.000	
Max		1000		1000		1000		3.000		3.000		3.000	
Mean		345.00		395.00		320.00		1.864		1.791		1.485	
		281.86		351.65		372.19		1.260		1.355		1.391	

**Table 4:** **Table 4:** Preintervention pairwise multiple comparisons of mean colony-forming unit/ml counts of *Candida albicans* in three groups (log_10_ values)

*Sl. no.*		*Comparison*		*t-value*		*p-value*	
1		Group A *vs* group B		0.106		0.916	
2		Group A *vs* group C		0.239		0.812	
3		Group B *vs* group C		0.138		0.891	

**Table 5:** **Table 5:** Postintervention colony-forming unit/ml count of *Candida albicans* in three groups - absolute and log^10^ values

		*Absolute values*						*Log_10_ values*					
		*Group A*		*Group B*		*Group C*		*Group A*		*Group B*		*Group C*	
Min		0		0		0		0.000		0.000		0.000	
Max		500		500		500		2.699		2.699		2.699	
Mean		165		150		190		1.436		1.109		1.268	
SD		166		201		220		1.215		1.268		1.310	

**Table 6:** **Table 6:** Analysis of variance of postintervention of mean colony-forming unit/ml count of *Candida albicans* in three groups

		*Sum of* * squares*		*df*		*Mean* * square*		*F*		*Sig.*	
Between groups		1.069		2.000		0.535		0.334		0.717	
Within groups		91.220		57.000		1.600					
Total		92.290		59.000							

**Table 7:** **Table 7:** Postintervention pairwise multiple comparisons of mean colony-forming unit/ml count of *Candida albicans* in three groups

*Sl. no.*		*Comparison*		*t-value*		*p-value*	
1		Group A *vs* group B		0.833		0.410	
2		Group A *vs* group C		0.421		0.676	
3		Group B *vs* group C		0.389		0.699	

**Table 8:** **Table 8:** Preintervention and postintervention comparison of change in *Candida albicans* colony-forming unit/ml count in three groups (values in log_10_)

				*Preintervention*				*Postintervention*				*Change*				*Significance of chance*			
*Sl. no.*		*Group*		*Mean*		*SD*		*Mean*		*SD*		*Mean*		*SD*		*t-value*		*p-value*	
1		A		1.86		1.26		1.44		1.21		0.43		0.72		2.654		0.016	
2		B		1.79		1.35		1.11		1.27		0.68		1.05		2.898		0.009	
3		C		1.46		1.39		1.27		1.31		0.22		0.66		1.464		0.160	

SD: Standard deviation

As far as change in mean CFU/ml of *C. albicans* was concerned, it was maximum in group B (0.68 ± 1.05) followed by group A (0.43 ± 0.72) and group C (0.22 ± 0.66). Except for group C, in both group B and group C, change was statistically significant (Table 8).

**Graph 3:**
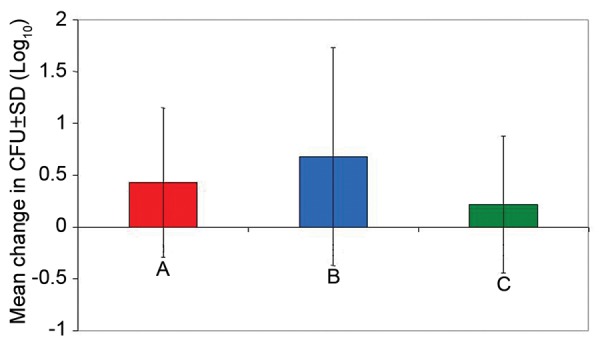
Pre- and postintervention comparison of change in *Candida albicans* colony-forming unit/ml count in three groups (values in log_10_)

In terms of change in *C. albicans* CFU/ml count, the exponential change in the three groups was 0.43 ± 0.72, 0.68 ± 1.05 and 0.22 ± 0.66 CFU/ml respectively groups A to C. Statistically significant results were obtained, maximum reduction was observed in group B (0.2% chlorhexidine digluconate rinse) followed by group A (probiotics) and least reduction was seen in group C (herbal rinse) (Graph 3).

## DISCUSSION

Oral cavity is colonized with microorganism soon after birth and forms an indigenous flora, which persists lifelong and prevents the growth of other microorganisms. *C. albicans* is present as commensal flora in 20 to 40% of healthy subjects and becomes the predominant flora in 60% of immunocompromised subjects.^11^

Daily oral home care helps in maintaining healthy oral environment and is an effective means for the prevention of oral diseases. Hence, pediatric dentists often recommend chemical adjuncts with routine toothbrushing, which provides unique and beneficial approach in the prevention and treatment of oral diseases. But injudicious use of antimicrobial agents disturbs normal microflora, favoring the establishment of opportunistic microflora.^10^

*Candida albicans,* though part of normal commensal flora, induces certain mycotic conditions like candidiasis secondary to oral dysbiosis. It is one of the most prevalent superficial mycosis (fungal infection) in pediatric patients. If left untreated it might serve an important port of entry for serious systemic fungal infections (deep mycosis).^12^ Furthermore, it was suggested that *C. albicans* displays many pathogenic factors, in that it is capable of adhering to various surfaces, interfering with the immunological system of the host organism as a dimorphic yeast and producing several catabolytes.^11^

With the advent of systemically administered antibacterial and antifungal agents, a hope for containment of these diseases occurred. But the recent reports of resistance to these agents dampened enthusiasm of dental professionals regarding their use and search for ideal oral rinse continued. Subsequently attention was focused toward an array of topical antimicrobials for the prevention of fungal overgrowth in the oral cavity.^13^ The current study is one such attempt in order to evaluate the effectiveness of probiotics in reducing the count of *C. albicans* in the oral cavity in comparison with commercially available and popular antibacterial products like chlorhexidine and herbal oral rinses.

Giuliana et al^12^ found that chlorhexidine is an effective means to inhibit candidal growth due to its ability to break the permeability barrier and leakage of cytoplasmic contents. Further in 2007, Torres et al^1^ revealed that even though chlorhexidine oral rinse dramatically reduces *C. albicans* counts, when patient discontinues treatment, intensity of colonization rises again. In our present study, 0.2% chlorhexidine digluconate had shown better antimicrobial effect than probiotics in reducing *C. albicans* counts after 1 week of intervention but there was no statistical difference in chlorhexidine and probiotic group, indicating probiotic oral rinse is equally effective.

Herbal oral rinse in our present study had minimum effect on *C. albicans* after 1 week of intervention. These findings were in contrast to observations documented by Lloyd et al^5^ who inferred that *A. indica* was quiet effective in suppressing candidal infections and found it nontoxic, nonmutagenic, immunostimulant, with antioxidant properties.

In our present study, *A. indica was* one of the ingredients. Apart from it, the other ingredients were Triphala which contains equal proportions of dry powder of *Terminalia chebula, Terminalia belerica* and *Emblica officinalis* and is extensively used in dentistry because it maintains gingival health and has anticariogenic property.^6^ *Mimusops elengi* has antiseptic and curative property; *Acacia catechu* extracts exhibit anti-inflammatory, antioxidant and antimicrobial properties; *Quercus infectoria* has the potential to generate herbal metabolites having anticarious activity; tulsi *(Ocimum sanctum)* has several scientific evidences to prove its antimicrobial, anti-inflammatory, anticarcinogenic, immunomodulatory properties;^14^ clove *(Caryophyllus aromaticus)* is widely used in dentistry due to its analgesic, local anesthetic, anti-inflammatory and antibacterial property.

Aboellil and Al-Tuwaijri^15^ concluded in their *in vitro* study that in controlled pH of 10, the inhibitory activity of natural (herbal) products against *C. albicans* was quite appreciable, whereas our present study was an *in vivo* study and pH might vary in trial subjects. The difference in our findings could also be attributed to shorter duration of study or difference in herbal preparation.

Antimicrobial agents act nonspecifically by reducing levels of both harmful and beneficial bacteria of oral cavity. Few microbes are bad but majority are beneficial to us called good bacteria or ‘probiotics’ and play a role in our general health and well-being. Probiotics provide a natural defense against bacteria though are harmful to teeth and gums, by utilizing natural beneficial bacteria of oral cavity, thus maintaining a balance of healthy microorganisms in the oral cavity. In our present study, a significant reduction in *C. albicans* count was observed in probiotics (Group A). Our results correlated well with research conducted by Dos Santos et al^16^ who suggested that probiotics act on candidal species by formation of IgA anti*-Candida* in oral cavity and probiotics mediated a mechanism of competition and interference in adhesion of *Candida* to epithelial cells. Elahi et al^13^ in an *in vivo* study observed similar findings as Stamatova et al,^8^ a significant reduction in *C. albicans* counts after oral administration of probiotics and their results were in accordance with our present study.

*Streptococcus uberis KJ2, Streptococcus oralis KJ3* and *Streptococcus rattus J* strains are susceptible to commonly used therapeutic antibiotics with no adverse effect. Zahradnik et al^17^ demonstrated that probiotic (Probiora3) was able to substantially affect the level of pathogens in saliva and subgingival plaque.

The results of the present study provided conclusive evidence that incorporation of probiotics oral rinse into dental hygiene products would be an attractive alternative to chemical additives for oral hygiene maintenance and well-being. However, a longitudinal study with larger sample size needs to be undertaken to evaluate definitive therapeutic mechanism of probiotic oral rinses.
